# Fake news detection in Urdu language using machine learning

**DOI:** 10.7717/peerj-cs.1353

**Published:** 2023-05-23

**Authors:** Muhammad Shoaib Farooq, Ansar Naseem, Furqan Rustam, Imran Ashraf

**Affiliations:** 1 Department of Computer Science, University of Management and Technology, Lahore, Pakistan; 2 Department of Software Engineering, University of Management & Technology, Lahore, Lahore, Pakistan; 3 Information and Communication Engineering, Yeungnam University, Gyeongsan si, South Korea

**Keywords:** Fake news detection, Ensemble learning, Machine learning, Urdu fake news

## Abstract

With the rise of social media, the dissemination of forged content and news has been on the rise. Consequently, fake news detection has emerged as an important research problem. Several approaches have been presented to discriminate fake news from real news, however, such approaches lack robustness for multi-domain datasets, especially within the context of Urdu news. In addition, some studies use machine-translated datasets using English to Urdu Google translator and manual verification is not carried out. This limits the wide use of such approaches for real-world applications. This study investigates these issues and proposes fake news classier for Urdu news. The dataset has been collected covering nine different domains and constitutes 4097 news. Experiments are performed using the term frequency-inverse document frequency (TF-IDF) and a bag of words (BoW) with the combination of n-grams. The major contribution of this study is the use of feature stacking, where feature vectors of preprocessed text and verbs extracted from the preprocessed text are combined. Support vector machine, k-nearest neighbor, and ensemble models like random forest (RF) and extra tree (ET) were used for bagging while stacking was applied with ET and RF as base learners with logistic regression as the meta learner. To check the robustness of models, fivefold and independent set testing were employed. Experimental results indicate that stacking achieves 93.39%, 88.96%, 96.33%, 86.2%, and 93.17% scores for accuracy, specificity, sensitivity, MCC, ROC, and F1 score, respectively.

## Introduction

The rapid dissemination of news through social media has made a significant impact on people due to the wide adoption of social media. According to the Pew Research Report 2021, 86% of adults in the United States (US) alone receive news from the internet, surpassing traditional news media (Shearer, 2021). Due to easy access to social media and its global wide use, the dissemination of fake, altered, or engineered content has become easier than before. As a result, individuals and groups attempt to contest and mislead society with the information they want. The phrase ’fake news’ became widely used on the internet and was declared a word of the year by the Collins dictionary in 2017 (Shu et al., 2020). Fake news can target individuals, groups, companies, as well as, governments and can potentially damage reputation, goodwill, and public perception. In addition, financial distress and turmoil can also be unleashed. There is a need for automated tools and techniques for detecting forged news because doing it manually would be inefficient and time-consuming (Jonathan et al., 2021; Riedl, 2019).

Fake news is engineered to deceive people and weaken public trust (Pierri & Ceri, 2019; Bozarth & Budak, 2020). To overcome this problem, an automated tool should be developed to check the authenticity or fakeness of news. Natural language processing (NLP) is an approach for handling text data that enables computers to interpret and interact with natural language (Wilks & Brewster, 2009). NLP facilitates the development of several applications, including text classification, question answering, machine translation, and much more (Church & Rau, 1995). Text classification has the most commonly utilized area in NLP problems being able to determine the semantic meaning of a sentence, word, or document (Manek et al., 2017).

Fake news detection is not easy and this task has several challenges. For example, the collection of the benchmark dataset and annotating it manually is a challenging task. This problem becomes more complicated with low-resource languages like Urdu for which a few online resources are available. Although there exist several approaches for Urdu fake news detection, they lack in several aspects. Such approaches do not use multi-domain data. Acquiring data from more domains allows for a more thorough evaluation of the performance of predictors. The performance of the models is better to be tested using data from a higher number of domains. Amjad et al. (2020) conducted a study to detect fake news with five domains. In addition, for dataset collection, some studies use Google Translate for English-to-Urdu translation but no manual verification is performed which reduces the scope of such models for real-world applications (Amjad et al., 2020; Akhter et al., 2021). Also, the number of samples in the dataset used in Amjad et al. (2020) is comparatively low due to which the models might not be trained and tested well. In previous works, fake news from five domains is considered with 900 samples only (Amjad et al., 2020).

This study aims at resolving these issues for Urdu fake news detection. In this work, a fake news classifier is developed to detect fake news from multi-domain data.

• An ensemble classifier is proposed for Urdu fake news detection. It uses an extra tree (ET) classifier and random forest (RF) as the base learners while logistic regression (LR) is used as the meta-learner.
• Stacking of features has been used by combining the feature vectors of preprocessed text and verbs extracted from preprocessed text. Feature stacking has not been employed before for the Urdu language, as per the best knowledge of the authors.
• A large corpus is collected from nine different domains which count to 4,097 news. It is manually annotated and is available publicly. Experiments are carried out using the term frequency-inverse document frequency (TF-IDF) and a bag of words (BoW) with n-grams using support vector machine (SVM), k-nearest neighbor (KNN), RF, ET, and LR.

**Figure 1:**
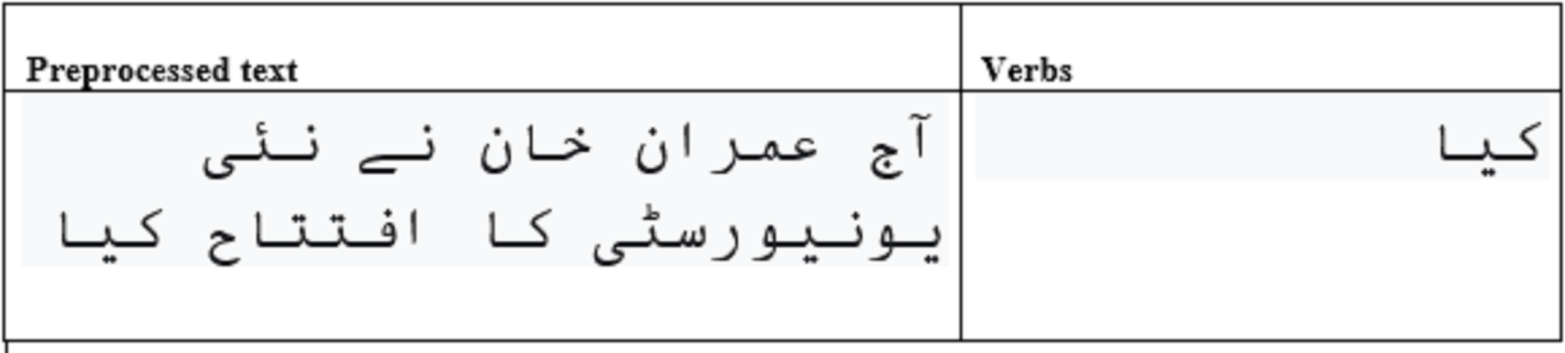
The verbs extraction from preprocessed text.

For feature stacking, firstly, the feature vector is generated using the cleaned text. Secondly, verbs from the cleaned text are obtained, and then the feature vector is computed for verbs. A sample of feature extraction of the verb is shown in Fig. 1. In the end, these two generated feature vectors are stacked into one feature vector.

The rest of this study is organized as follows. ‘Related Work’ comprises related work where previous studies for fake news detection in the Urdu language have been discussed. ‘Materials and Methods’ consists of the proposed method where architectural look and explanation of the method for fake news detection. It is followed by a discussion of experimental results. In the end, the conclusion is given.

## Related Work

Many machine learning-based approaches have been developed for the identification of fake news in the Urdu language. Maaz Amjad conducted two studies for fake news detection in the Urdu language. In Amjad et al. (2020), the benchmark dataset contributed overall 900 samples with 500 samples for Urdu real news and 400 samples for Urdu fake news. However, this data is considered small in terms of domains and samples. In real-time, the news might occur from outside the domains with fewer samples where the model might have lacked the identification of fake news in the Urdu language. Another limitation of this study is that researchers have generated fake versions of real news, which might not be a suitable method. The second study employed by Amjad, Sidorov & Zhila (2020) for fake news detection in the Urdu language generated different datasets using methods such as augmented, augmented-downsized, and machine-translated versions of the original dataset with the benchmark dataset. However, just comparisons of approaches have been carried out, rather than new contributions.

Akhter et al. (2021) utilized an ensemble approach to detect fake news in the Urdu language. This study has contributed a new dataset along with experimentation on the benchmark dataset created by Amjad, Sidorov & Zhila (2020). However, this study lacks the ability to produce a highly accurate model for the identification of fake news in the Urdu language. In addition, this study has also used an English-translated dataset in Urdu language using Google Translate without manual verification. This study uses the data from a total of five domains only and its scope is small.

Lina, Fua & Jianga (2020) proposed a deep learning model called CharCNN-RoBERT for fake news detection in the Urdu language. The study uses the dataset developed by Amjad et al. (2020), which contains 900 samples for experiments. The study used the combination of RoBERTa, charCNN, and pre-training along with word and character n-grams for fake news detection in Urdu. Results indicate that using a combination of RoBERTa, charCNN, pre-training, and label smoothing, an accuracy of 0.90 is possible for Urdu fake news detection. Similarly, the authors adopt word and character n-grams to train machine learning models for fake news detection in the Urdu language (Balouchzahi & Shashirekha, 2020). Additionally, word embedding vectors are utilized for training deep learning models for the same purpose. An accuracy of 0.79 and an average F1 score of 0.78 is obtained using a machine learning-based ensemble approach.

The above-discussed studies have several limitations. First, the dataset has been converted from English to Urdu but manual verification is not carried out. Secondly, the number of samples in the dataset is smaller and proper evaluation of the models is not possible using a smaller number of samples. The dataset used in previous studies was limited to fewer domains. Previous studies were not able to cover different dimensions due to fewer domains. This study incorporates nine different domains for data acquisition from various dimensions.

The limitation of existing studies for Urdu fake news identification and comparative literature of studies are elaborated in Table 1. This study aims at resolving these issues for Urdu fake news detection. This study proposes a new dataset along with a classifier developed for Urdu fake news identification from the multi-domain dataset. Previous studies utilize 900 fake news samples from five domains. The proposed work has contributed by increasing the number of news up to 4,097 from nine domains. In addition, manual verification has been carried out from Google-translated news, which is not a 100% trusted tool for effective translation. The dataset has been acquired from Kaggle and used in a study (Ahmed, Traore & Saad, 2017) for English fake news detection. Manual verification has been carried out against each news to overcome this problem. Feature engineering techniques like TF-IDF and BoW have been used with word and character level n-grams.

**Table 1:** Analytical summary of the discussed research works.

Study	Features	Methods	Samples	Limitations	Domains	MT manually verified
Amjad et al. (2020)	Word, character and functional n-grams	AdaBoost, LR, SVM, RF, MNB, BNB, DT	900	Small dataset 900 news. Fake news is created based on real news.	5	–
Amjad, Sidorov & Zhila (2020)	Word, character and functional n-grams	SVM, AdaBoost	400	Machine translated dataset. No manual verification.	–	No
Akhter et al. (2021)	Character tri-grams, BoW and Information Gain	NB, DT, SVM	2000	Machine translated dataset. No manual verification. Un-optimized results	–	No
Lina, Fua & Jianga (2020)	Word and character n-grams	RoBERTa, charCNN	900	No dataset is contributed.	–	–

## Materials and Methods

**Figure 2:**
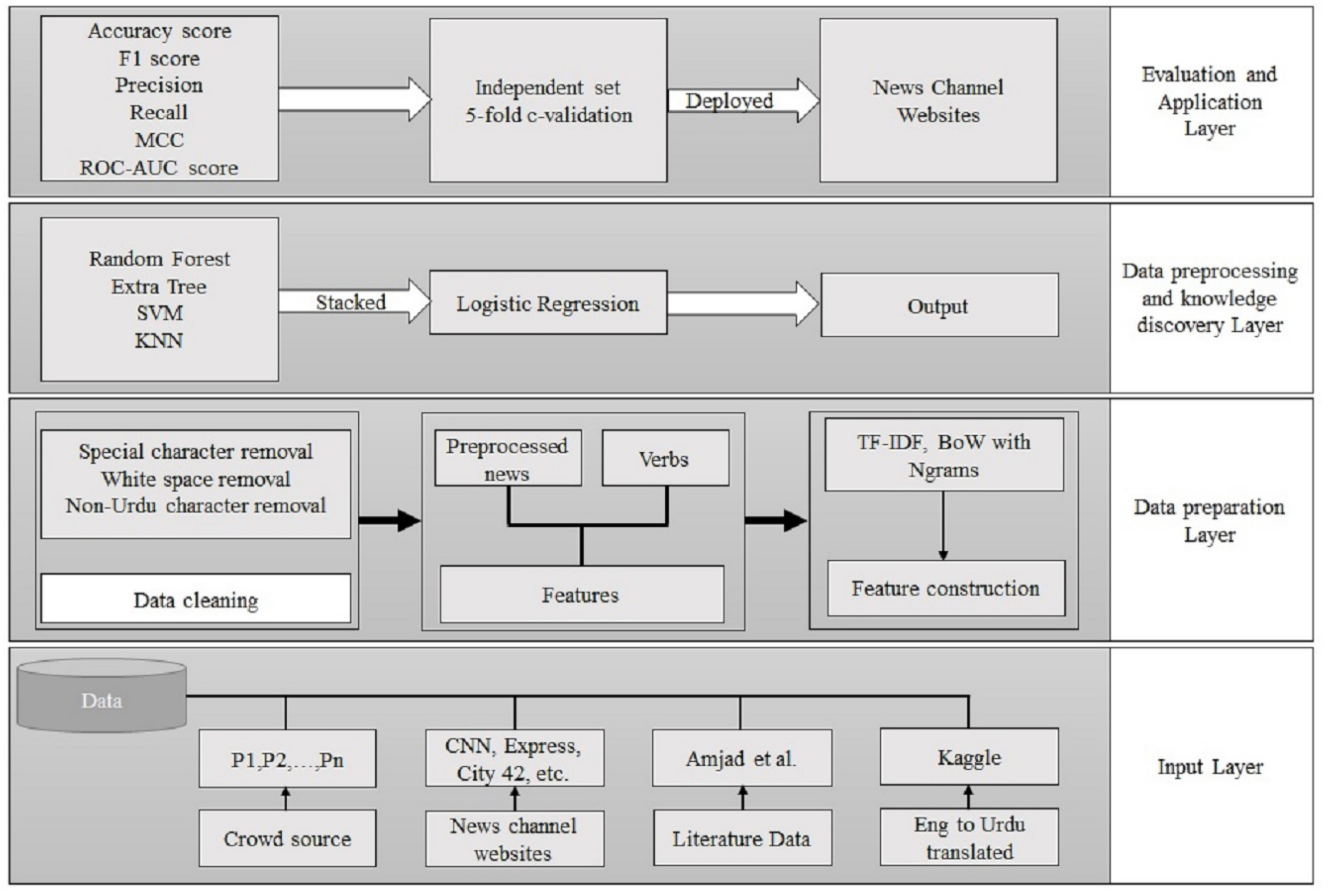
The workflow of the proposed approach.

This part discusses the architecture of the adopted methodology. Bagging and stacking ensemble approaches are also discussed in this section. Figure 2 shows the workflow of the adopted methodology for fake news detection. It comprises an input layer, a data preparation layer, a data preprocessing strategy and knowledge discovery layer, and an application layer. The input layer obtains the data from crowdsourcing, news channel websites, literature available data, and data converted from English. In the next layer, data cleaning and feature construction are performed. The third layer consists of model building for the fake news model. In the last layer, the trained model is deployed for fake news detection.

### Input layer

The first layer comprises data acquisition from different sources like news websites, data from existing studies, and machine-translated datasets. The real news dataset was acquired from various sources *i.e.,* literature dataset from Amjad, Sidorov & Zhila (2020), and different news channels websites such as BBC Urdu News, Dawn News, and City 42 News collected from May 2020 to March 2021. There is no proper repository available for fake news in the low-resource Urdu language. Different sources were used for Urdu fake news data such as Vishvas news; fake news data was attained from the English fake news dataset and converted into Urdu with manual verification. Similarly, Urdu fake news dataset from a published article (Amjad, Sidorov & Zhila, 2020) and crowdsourcing was also obtained.

### Process of data annotation

Three independent annotators have been employed to label the acquired news. These experts have been hired with the following expertise for effective data collection:

• Familiar with social media,
• Urdu native speakers,
• Having at least a Master’s degree within the domain,
• Isolated from any social media or news channel job,
• Having experience in data annotation.

#### Real news acquisition

The dataset has been annotated manually for real news and the following factors have been taken into consideration for real news acceptance criteria.

• News acquired from an authentic website,
• Authentic news channel,
• Authentic newspaper.

The sources used to obtain real Urdu news have been listed in Table 2.

#### Fake news acquisition

Urdu is a low-resource language; there is no repository available for Urdu fake news. The Urdu fake news dataset has been collected from three major sources: (i) websites, (ii) crowd-sourcing, and (iii) fake news datasets for the English language published in a research article (Ahmed, Traore & Saad, 2017). The published fake news data from the English language has been converted into the Urdu language with manual verification. If any news does not make sense, either the news has been removed or the news has been corrected by consulting the official source. Lastly, the fake news dataset was acquired from a published research paper by Amjad et al. (2020) for Urdu fake news detection. Other sources for fake news data are listed in Table 3.

**Figure 3:**
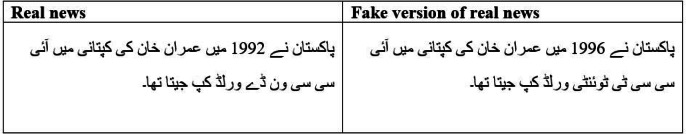
Real *vs* fake news.

The crowd-sourced professionals are directed to generate random fake news, which leads to an unbiased dataset. An example of opted strategy is shown in Fig. 3. In the previous study (Amjad et al., 2020), the crowd-sourced professionals were asked to generate fake news by changing the minor content of real news, which leads to a biased dataset. This study, however, does not generate fake news and considers only those which are found in existing datasets or obtained from other sources listed in Table 3.

**Table 2:** Acquisition sources for real news.

News sources	URL
BBC Urdu News	www.bbc.com/urdu
Dawn News	www.dawnnews.tv
City 42 News	www.city42.tv
Express Newspaper	www.express.com.pk/

**Table 3:** Acquisition sources for fake news.

News sources	URL
Vishvas news	www.vishvasnews.com/urdu
Fake news dataset	https://www.kaggle.com/datasets/clmentbisaillon/fake-and-real-news-dataset
Urdu fake news dataset	https://github.com/MaazAmjad/Datasets-for-Urdu-news
Crowdsources	–

Regarding the efficacy of dataset annotation from three annotators, we determined inter-annotator agreement (IAA) using Cohen’s Kappa coefficient, which is a statistic evaluator used to determine whether two annotators could be relied upon. The evaluation leads to a 92% overall score.

**Figure 4:**
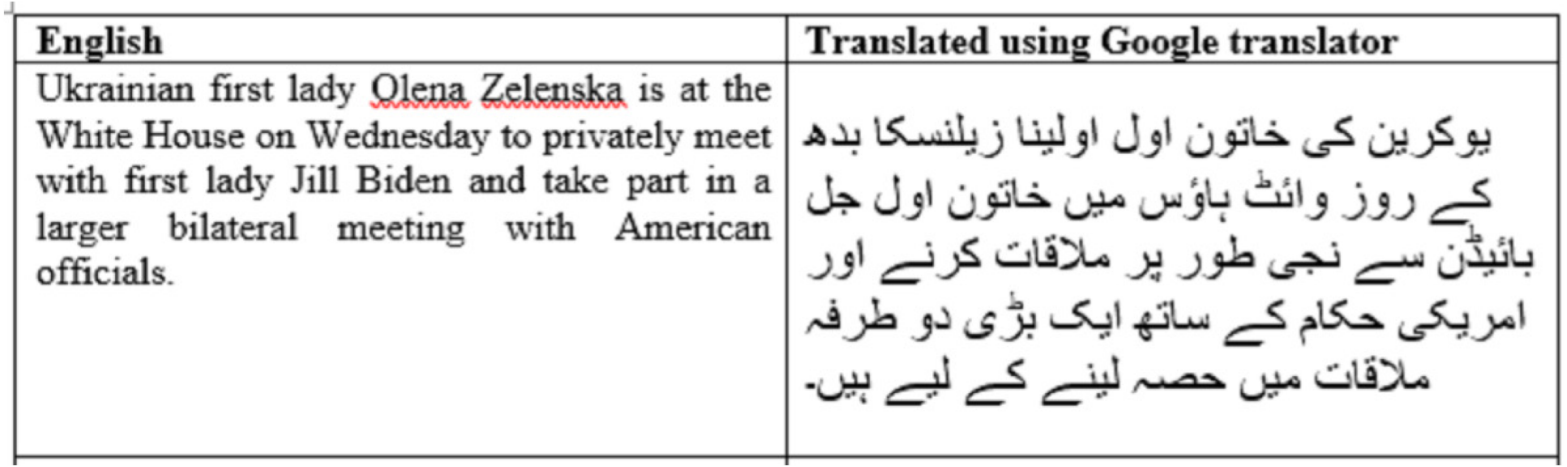
A news sample that has been discarded after manual verification.

Lastly, an English dataset from Kaggle was selected for machine-translated news. Instant Scrapper was used to acquire real news data from different websites. The fake news data was collected from the English language, then converted into the Urdu language, and translated news was manually checked (Ahmed, Traore & Saad, 2017). If any news does not convey meanings properly, either the news was removed or the sequence or wording is manually corrected. Figure 4 shows a news sample which has been discarded.

We collected fake and real news over the last three years using various tools and methodologies. There are 4,097 records in the corpus, with 2,455 false news and 1,642 true news. Even though researchers examined numerous types of information while creating a corpus, the annotation process may vary slightly. Before employing the dataset, it was double-checked. Some previous studies focused on specialized areas, such as politics, and developed models for a single dataset. This method suffers from dataset biases and will likely perform badly on news from another domain. To resolve such issues, this study collected the news from nine different categories. Table 4 shows the domains for which news have been collected, as well as, the number of real and fake news for each domain.

### Data preparation layer

In the second layer, collected data was prepared because raw data in real life comprises noise and redundant information. It increases the processing time and has a negative impact on model accuracy. The data must be clean and consistent before being fed into the machine learning model.

#### Data cleaning

The collected dataset comprises numerical values, special characters, and a uniform resource locator (URL). As some portion of the dataset was acquired from news channels websites using web scraper, the news contains special characters and other language words. To ensure data quality, it is critical to do various preprocessing stages. To create high-quality data, we reviewed it manually and removed any news that does not make sense. After this process dataset is tokenized. Tokenization is the process of separating all text by white space and removing noise such as numerical characters, special characters, and URLs. Punctuation in the text serves as a grammatical context and does not add much to the interpretation of a sentence. Commas, full stops, and other punctuation marks are used in the news, so finally punctuation has also been removed from the data.

**Table 4:** Class-wise real and fake news for the collected dataset.

Domain	No. of records	Real news	Fake news
Health	519	189	330
Business	450	190	260
Sports	540	178	362
Technology	380	142	238
Showbiz	400	168	232
Politics	609	259	350
Science	559	254	305
Crime	190	93	97
Travel	450	169	281
Total	4,097	1,642	2,455

#### Features

Features play a key role in machine learning in text classification. For this study, we employed verbs extracted as features from preprocessed news and raw cleaned text features based on preprocessed news. First, we computed features on preprocessed news; then from preprocessed news, verbs were extracted as features. Lastly, feature vectors were generated from these two features and combined to construct one feature vector. This feature vector was fed to the model for the prediction of fake and real news.

#### Feature construction

To compute features two main techniques were employed including TF-IDF and BoW. The word level and character uni-grams, bi-grams, and tri-level feature vectors were used with both features on the preprocessed news and verbs extracted from preprocessed news.

TF-IDF is a statistical technique, which is used to determine how important a word in a document or corpus is Liu et al. (2018). The term frequency refers to the number of times a term appears in a news. The frequency of a term in news reflects its significance. Term frequency transforms each term in the news as a matrix with the number of news in the rows and the number of distinct words in the columns. Document frequency comprises all news having a feature and reflects the commonality of the feature. The weight of a feature is determined by the inverse document frequency (IDF), which minimizes the weight of a feature if the feature’s occurrences are dispersed throughout all news. To convert news into numbers first BOW is employed. In the proposed approach, the frequency-based count is employed. It counts the occurrence of a word in each news and gives the frequency with a fixed length matrix.

NLP can make effective use of n-gram data to understand a specific pattern in text data (Rizwan, Shakeel & Karim, 2020). To create features from the preprocessed news, word-based n-gram, and character-based n-gram are employed, which assists the model in predicting real and fake news. We employed character levels n-grams ranging from unigram (*n* = 1) to tetra-gram (*n* = 4) in this work. Unigram (*n* = 1) and bigram (*n* = 2) techniques are utilized to train the model on word-level n-gram. This characteristic was used because it provides structural information and refines the words in a more relevant way.

### Data processing and knowledge extraction layer

In this layer, a fake news classifier is developed using different machine learning models. Ensemble-based approaches like bagging and stacking are employed. Classifiers for each approach are explained here briefly.

The methodology part explains the architecture of different machine learning algorithms which are utilized to improve the fake news prediction model. In this study, the machine learning algorithms LR, RF, and ET were employed. Each algorithm was individually experimented with to detect fake news in the Urdu language. After that, the algorithms were stacked together to make a more accurate model. The explanation of each model is described below.

#### Random forest

RF belongs to the bagging family and uses a bootstrapping technique for sample distribution (Attique et al., 2020). First, the model creates sub-datasets consisting of fake and real news consisting of sampling with replacements. Each subset has an equal-sized distribution of fake and real news. The model receives the news in the form of a feature vector with the label for training purposes and decision trees created with random best-split nodes. A test instance is fed to all weak learners, and the class prediction is made with the majority vote.

#### Extra tree

ET is another bagging method that receives the news without replacement along with the label for training purposes. ET generates many sub-datasets with equal-sized news in each subset. When a query instance is fed to the model, this instance is given to all weak learners and class prediction is made as per the majority vote.

#### Stacking

**Figure 5:**
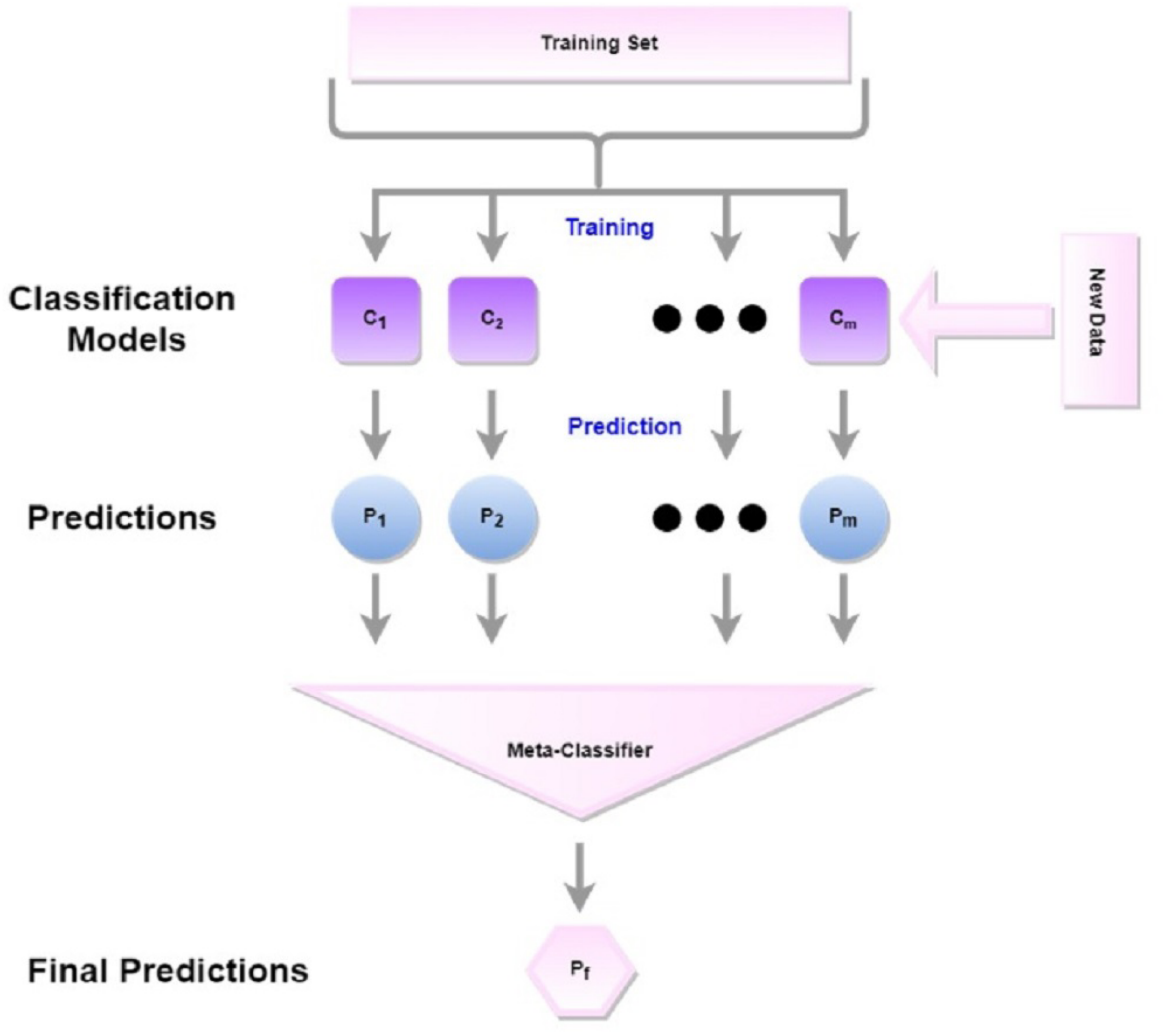
The workflow for stacking.

Stacking is an ensemble approach where classifiers construct predictions from each model for each piece of news and combine those predictions to construct a new dataset, as shown in Fig. 5. Based on the individual performances of these classifiers, the top two classifiers, RF and ET have been selected as base learners. The generated prediction from RF and ET against each news forms a two-dimensional vector. This 2D vector has been split into train and test and has been given to the final learner.

When a model receives the news in the form of a feature vector, the weights are multiplied by input data. After acquiring the sum of each news source, the sum is given to the sigmoid function. The sigmoid function leverages the sum to reduce the scope of probability space ranges from 0 to 1 (Zhang, Yang & Zhang, 2018; Tolles & Meurer, 2016) with a 0.5 threshold value. *Z* represents the sum for each number and *Y* represents the sigmoid function for the following equations.

(1) \begin{eqnarray*}z=({w}_{1}{x}_{1}+{w}_{2}{x}_{2}+,\ldots ,{w}_{n}{x}_{n}+b)\end{eqnarray*}

(2) \begin{eqnarray*}Y= \frac{1}{1+{e}^{-z}} .\end{eqnarray*}

#### Support vector machine

The news is sent to the SVM classifier, which constructs the best-separating hyperplane. The line where SVM separates news of specified classes is known as the decision boundary. To choose the optimum model, we must first identify the decision boundary of the linear kernel. We used the following equation to obtain the boundary line

(3) \begin{eqnarray*}y=b+{\omega }_{1}\ast {x}_{1}+{\omega }_{2}\ast {x}_{2}+,\ldots ,\end{eqnarray*}

where *x*_1_ is the news feature, *ω*_1_ indicates the weight stated in the coefficient, and *b* is the slope.

We can use the hyperplane to make predictions once it has been constructed. The following is the formulation of the hypothesis function *h*

(4) \begin{eqnarray*}h(x)= \left\{ \begin{array}{@{}ll@{}} \displaystyle 1 &\displaystyle \text{if}\omega \ast x+b\geq 0\\ \displaystyle -1 &\displaystyle \text{if}\omega \ast x+b\lt 0. \end{array} \right. \end{eqnarray*}

When a new instance is introduced, it is assigned to the negative class if its score is less than 0, and the positive class if it is higher than 0. In SVM, several hyperplanes are possible. By increasing the distance between the hyperplanes of both classes, the performance of SVM can be improved.

#### K-nearest neighbor

KNN is an instance-based classifier where news is divided into training and testing sets for prediction with five neighbors. Each test vector is given to KNN, which computes the feature vector distance. The model sorts five news with the minimum distance against test news. The majority of vote prediction from five news is assigned to test news. This process is repeated for each test set. This study uses the Minkowski distance for KNN. Table 5 provides the complete list of hyperparameters used in this study.

### Application layer

The final layer corresponds to the application layer, which comprises the online platform, where the developed models are deployed. Platforms like social media platforms and news channels websites are used in this study.

### Evaluation measures

Several evaluation measures were used to evaluate the quality of the model’s performance. This study uses parameters like accuracy, specificity, sensitivity, and Mathew’s correlation coefficient.

The accuracy score is a fundamental metric for evaluating a model’s performance. The accuracy formula is stated below.

(5) \begin{eqnarray*}Accuracy= \frac{TP+TN}{TP+TN+FP+FN} .\end{eqnarray*}

Specificity was employed to provide a quantitative assessment of the model’s accuracy for predicting negative cases. The formula of specificity has been provided below.

(6) \begin{eqnarray*}Specificity= \frac{TN}{TN+FP} .\end{eqnarray*}

Another criterion is sensitivity, which indicates the model’s ability to accurately identify positive class samples. The formula of sensitivity is given below.

(7) \begin{eqnarray*}Sensitivity= \frac{TP}{TP+FN} .\end{eqnarray*}

**Table 5:** Hyperparameters for machine learning models.

Model	Hyperparameters
RF	N_estimators=100, min_sample_split=2, max_feature=’sqrt’
KNN	n_neighbors=5, metric=’minkowski’, p=2
LR	tol=0.0001, *C* = 1.0, solver=’lbfgs’
SVC	C=1.0, kernel=’rbf’, degree=3
ET	N_estimators=100, min_sample_split=2, max_feature=’sqrt’
Stacking	gamma=’scale’, Base_estimator=ET and Rf, final_estimator=LR

The F1 score is a measure of a classifier’s accuracy that considers both precision and recall. To provide a fair evaluation of a classifier’s performance, it takes into account both the number of true positives as well as false positives and negatives. A high F1 score indicates that a classifier has both high precision and high recall, while a low F1 score suggests poor performance.

(8) \begin{eqnarray*}F1=2\times \frac{Precsion\times Recall}{Precision+Recall} .\end{eqnarray*}

A more dependable and consistent statistical and robust metric is the Matthews correlation coefficient, which only produces a high score if the prediction performance is good in each of the four quadrants of the confusion matrix (Chicco & Jurman, 2020). The formula of MCC is given as

(9) \begin{eqnarray*}MCC= \frac{TN\times TP-FN\times FP}{\sqrt{(FP+TP)(FN+TP)(FP+TN)(FN+TN)}} .\end{eqnarray*}

A classifier’s capacity to distinguish between positive and negative classes is indicated by the ROC AUC score. It is calculated as the area under the receiver operating characteristic (ROC) curve, which plots the true positive rate against the false positive rate. A high ROC AUC score indicates a classifier with high accuracy in identifying positive and negative instances.

## Experiments and Results

This section comprises experiments and results obtained from the dataset. Several experiments are performed for Urdu fake news detection.

### Experiments

The dataset is prepared using data cleaning and feature construction steps discussed in the above sections. The overall number of words and vocabulary in the corpus is shown in Table 6.

**Table 6:** Description of the dataset regarding number of words and vocabulary.

Data	No of words
Clean corpus	316,355
Vocabulary	23,222

N-grams with word and character were utilized in this study and uni-gram, bi-gram, tri-gram, and tetra-grams were employed. For word n-grams, only uni-gram and bi-grams are employed. Due to the large corpus, the word n-grams reached over two lac features for bi-gram. The previous study (Amjad et al., 2020) suggested that character n-grams work better in the Urdu language as compared to word n-grams. The range of generated n-grams is given in Table 7.

**Table 7:** Character n-grams for the dataset.

Char n-gram combinations	Min range	Max range	No of features
(1,1)	1	1	36
(1,2)	1	2	2,043
(1,3)	1	3	18,276
(1,4)	1	4	91,901

### Results

Three sets of experiments are carried out to evaluate the performance and robustness of the model. The results for each of these experiments are discussed here.

#### Independent set testing

A well-known approach to evaluate the performance of the classifier with hidden data is independent set testing. For this assessment, the data is usually divided into two groups. The first section relates to the training set, which contains input and output pairs that are fed to the model to learn effectively. The second part consists of a test set where just input features are passed and labels are hidden. The model needs to predict the relevant class fake or real according to given features. The scores for independent set testing are shown in Table 8.

**Table 8:** Results using independent settings with TF-IDF.

Model	Accuracy	Specificity	Sensitivity	MCC	F1 score
RF	91.05	81.94	97.15	81.51	89.36
LR	89.1	83.16	93.08	77.18	87.80
ET	90.81	81.38	97.15	81.02	88.85
Stacked	93.82	93.5	94.49	86.10	93.17
KNN	78.45	80.17	85.07	54.62	77.20
SVM	89.59	81.95	94.71	78.26	88.96

Table 8 shows the experimental results using TF-IDF features. Results indicate that all models perform well except for KNN which shows a 78.45% accuracy. The stacked-based approach has shown the highest accuracy and MCC score which are 93.82% and 86.10% for Urdu fake and real news detection. RF, ET, and LR show MCC scores as 81.51%, 81.02%, and 77.18%, respectively.

**Figure 6:**
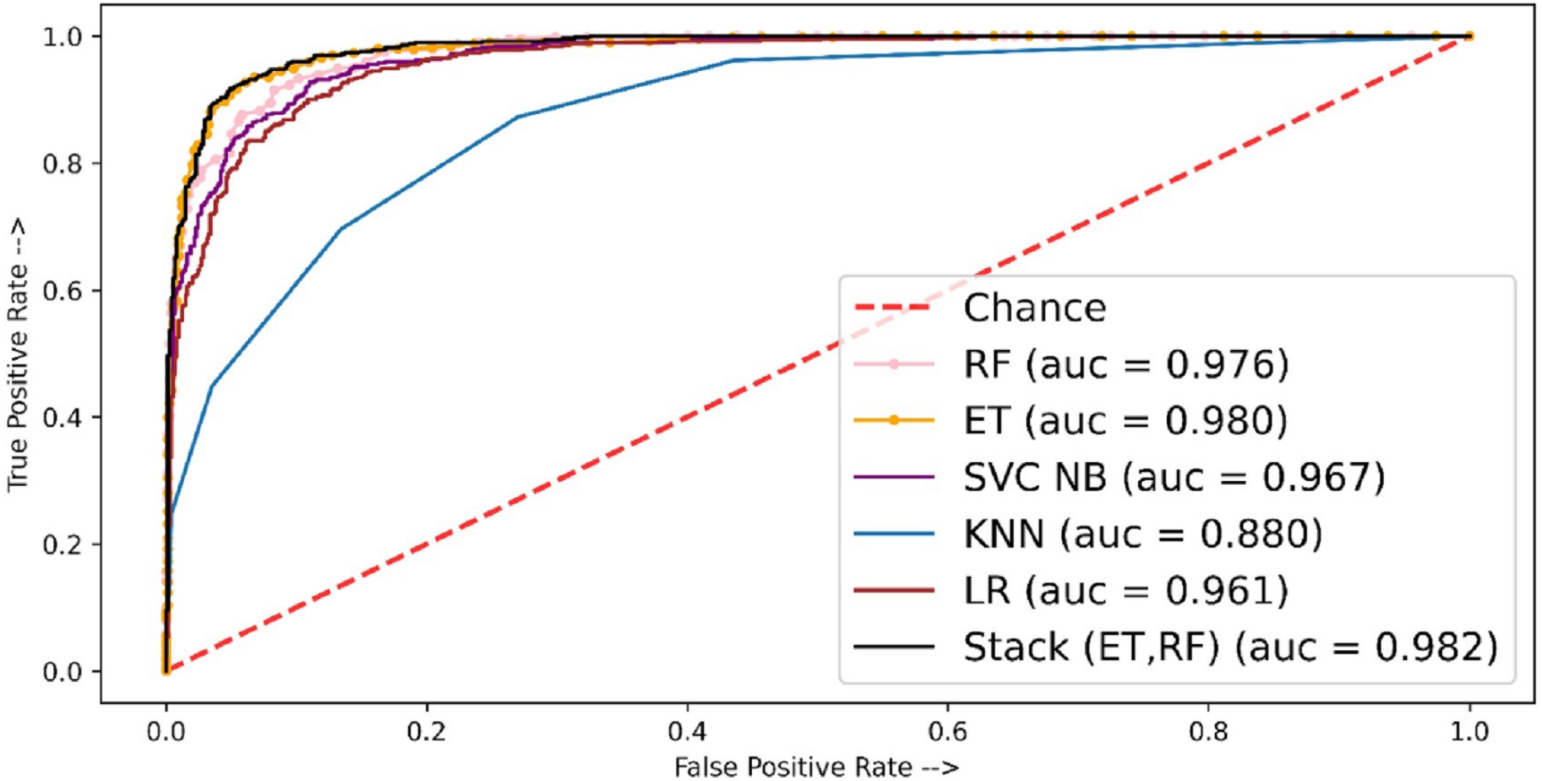
ROC-AUC plot for independent set using TF-IDF.

The performance of the models in terms of the ROC-AUC curve is shown in Fig. 6. Here the least ROC-AUC curve was provided by the KNN while the highest ROC-AUC curve was obtained by the proposed stacked model using TF-IDF features.

Table 9 shows the results achieved from machine learning models using the BoW features. Results indicate that the performance of the models is slightly degraded when used with the BoW features. The BoW provides the count of the terms in the corpus and does not record the importance of rare terms. Although often BoW produced better results than complex models, TF-IDF, which records the weight of important terms, produced better results in this study for Urdu fake news detection. Of the employed models, the stacked model tends to show better performance.

#### Cross validation

A cross-validation is a testing approach that differs from self-consistency and independent testing. Because all data is employed for training and the same data is used for testing, the predictor does not predict the unknown data in self-consistency. This gap directs the usage of independence set testing, which allows the predictor’s performance to be tested using unseen data. Nonetheless, because independent set testing is carried out on data that is randomly spread, a considerable percentage of the data may be missing. To address this issue, cross-validation was developed.

Cross-validation is a complete test that is done across all samples. It separates the data into discrete k-folds of a given length. However, in the previous studies, k was given a value of 5 or 10. *K* = 5 indicates that the data will be separated into five sections with an equal class ratio in each fold, each section with an equal amount of samples. The first fold is left out, while the remaining four will be employed for training and the left fold for testing. The second iteration will employ the second fold as a test set and the remaining four as a training set. The operation will be continued until the number of folds reaches k. Each fold’s accuracy is measured, and the final average will be determined as the final accuracy.

**Figure 7:**
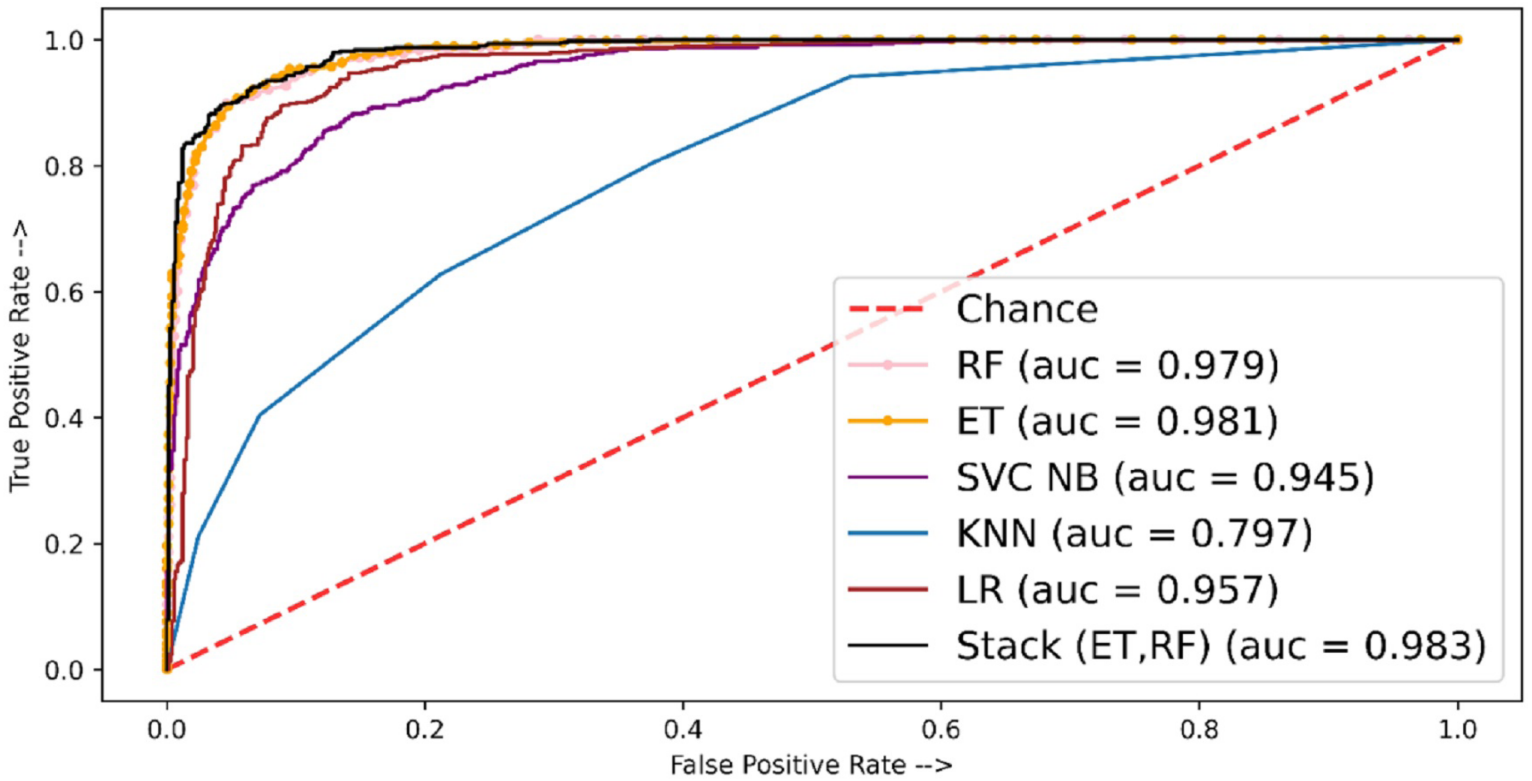
ROC-AUC plot for 5-fold cross-validation using TF-IDF.

Table 10 shows the experimental results of fivefold cross-validation where the stacked approach shows better performance as compared to individual classifiers. The stacked-based method showed the highest MCC score of 84.76 whereas other individual classifiers ET, RF, and LR showed MCC scores of 81.75, 80.08, and 77.45, respectively. Figure 7 shows the AUC ROC curve using TF-IDF features.

**Table 9:** Results using independent settings with BoW.

Model	Accuracy	Specificity	Sensitivity	MCC	F1 score
RF	91.38	84.18	96.20	82.05	91.60
LR	89.76	85.80	92.40	78.59	89.29
ET	92.28	85.60	96.74	83.94	91.40
Stacked	92.84	92.29	93.21	85.18	92.33
KNN	77.51	79.10	85.70	53.10	76.13
SVM	88.34	80.51	95.31	77.82	88.16

**Table 10:** 5-fold cross-validation results using TF-IDF.

Model	Accuracy	Specificity	Sensitivity	MCC	F1 score
RF	90.38	81.12	96.58	80.08	91.23
LR	89.21	81.97	94.05	77.45	89.87
ET	91.09	80.76	98.00	81.75	91.83
Stacked	92.68	88.06	95.80	84.76	92.30
KNN	78.12	81.25	85.22	55.73	77.07
SVM	91.43	85.11	92.77	80.82	88.53

For fivefold cross-validation, the ROC-ACU score was calculated for each fold of each classifier; then mean is taken for each classifier. For fivefold cross-validation experiments, ET shows the best performance with a 0.98 ROC-AUC score as compared to other models.

Table 11 presents the results obtained using BoW features for fivefold cross-validation. Results suggest that stacked based classifier outperformed individual models with an 85.08 MCC and 93.05% F1 score. Results attained using MCC from fivefold cross-validation for RF, LR, and ET were 83.33, 76.23, and 83.44, respectively.

**Table 11:** Fivefold cross-validation results using BoW.

Model	Accuracy	Specificity	Sensitivity	MCC	F1 score
RF	92.01	86.72	95.56	83.33	91.23
LR	88.54	82.71	91.27	76.23	87.34
ET	92.04	85.50	96.41	83.44	92.50
Stacked	92.85	88.67	95.64	85.08	93.05
KNN	79.50	82.45	86.72	56.67	79.20
SVM	90.54	84.51	95.87	79.87	89.75

Two types of testing were employed, including independent testing and k-fold cross-validation with five folds. The results for different models vary slightly in regard to independent testing and cross-validation testing, but the stacked model tends to show better performance than individual models.

**Figure 8:**
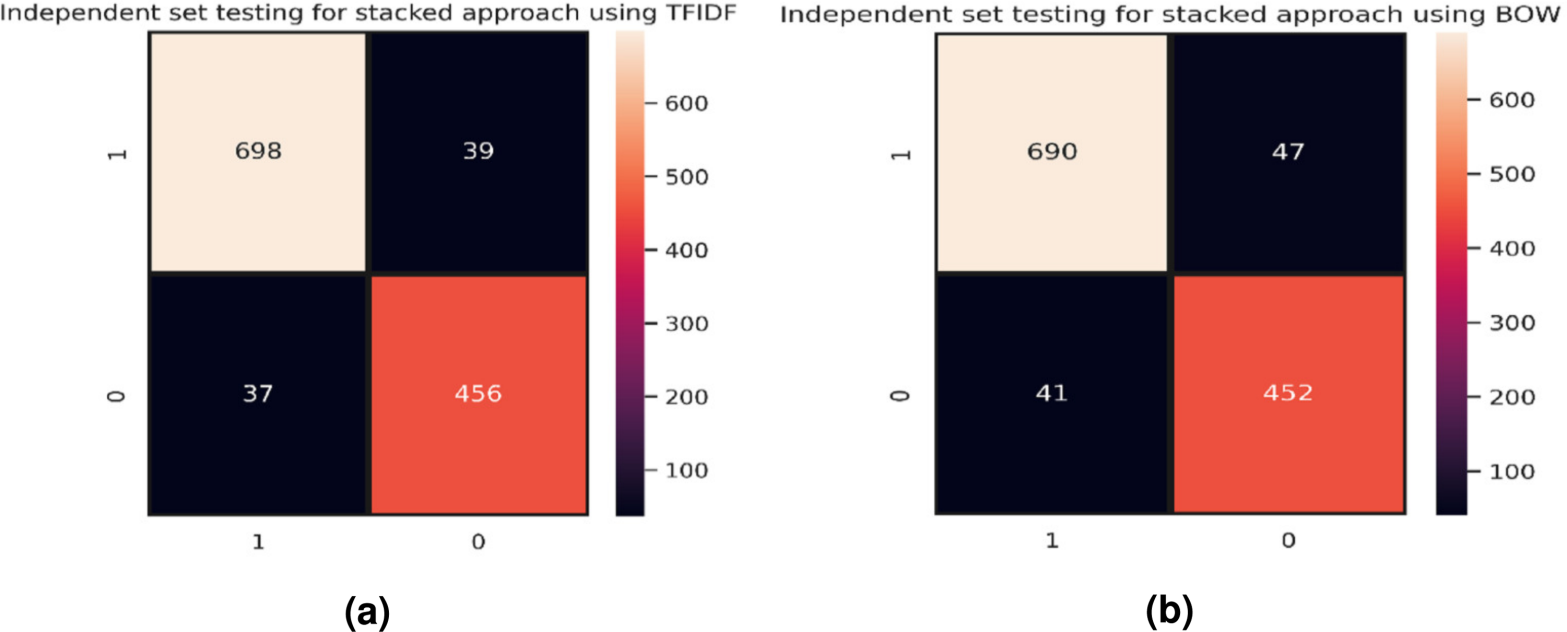
Confusion matrices, (A) stacking approach using TF-IDF, and (B) stacking approach using BoW.

Figure 8A shows the confusion matrix for the best model for independent set testing based on TF-IDF feature vectors where the stacked model has outperformed other classifiers. Figure 8B represents the best results achieved for independent set testing using the BoW feature vector. The confusion matrix is for the stacked approach as it shows the best results.

For corroborating the performance of the proposed approach, the results of the proposed approach are compared with state-of-the-art existing models for Urdu fake news detection. For a fair comparison, the models from existing studies (Amjad, Sidorov & Zhila, 2020; Amjad et al., 2020; Akhter et al., 2021) were implemented using the dataset collected in the current study, and performance is compared. Performance comparison results are given in Table 12. Results suggest that the proposed approach shows significantly better results as compared to existing studies. We tested the model with a robust measure called MCC as it considers both classes for evaluation. The proposed model shows the highest ROC-ACU of 0.98 as compared to the previous best study which was 0.95.

**Table 12:** Performance comparison with existing approaches.

Ref.	Accuracy	Specificity	Sensitivity	MCC	ROC	F1 score
Amjad, Sidorov & Zhila (2020)	84.23	93.20	82.30	75.34	0.940	84.30
Amjad et al. (2020)	83.48	91.34	83.21	74.38	0.956	87.37
Akhter et al. (2021)	81.85	89.33	84.37	70.37	0.927	82.72
Current study	93.82	88.96	96.33	86.20	0.983	93.17

### Discussions

In recent studies on Urdu fake news detection, limited domains are considered for fake news detection with a smaller corpus. Manual verification of Google-translated English news is also not carried out which raises questions regarding the efficacy of these models.

This study aims to solve these issues for the low-resource Urdu language. We increased the size of the dataset as compared to previously available data. The collected dataset consists of nine domains such as health, business, sports, technology, showbiz, politics, science, crime, and travel, and contains a total of 4,097 news. The collected dataset was cleaned by removing special characters, white spaces, non-Urdu characters, and stop words. For stop word removal, a previous study (Amjad et al., 2020) suggested that the removal of words in the Urdu language decreases the performance of the model. We removed the stop words and performance decreased, so for further experiments stop words have not been removed. Afterward, two types of features have extracted; first, the preprocessed text is converted into a feature vector, second, the verbs are extracted from the preprocessed text and finally, the two features are combined. For feature computation, TF-IDF and BoW with word level and character n-grams were employed.

Machine learning classifiers such as KNN, RF, ET, SVM, LR, and stacking were utilized. The ensemble method normally shows better results than individual classifiers. We stacked RF and ET as the base learners and LR was employed as the final learner. For evaluation purposes, metrics such as accuracy score, specificity, sensitivity, MCC score, F1 score, and ROC-AUC score were utilized to check the robustness of the model. The results indicate that the stacked model performs better than individual models for Urdu fake news detection. Performance comparison with existing studies confirms these results.

## Conclusion

Urdu is a low-resource language and no dedicated repository is available regarding real and fake news detection. Existing studies performed experiments with smaller datasets and multi-domain news is not very well investigated. This study is a multi-objective attempt to overcome these limitations. The first contribution is a large dataset of 4,097 news from nine different domains compared to five domains in previous studies. Data was collected from various online platforms like news websites, and existing articles, as well as translated from English to Urdu news. Manual annotation and verification were performed to ensure the correctness of real and fake news. The second contribution is to obtain higher accuracy for fake news detection. An ensemble classifier is proposed in this study that comprises RF, ET, and LR. In addition, the stacking of verbs extracted from the preprocessed text and preprocessed text has been utilized to form a combined feature vector using TFIDF and BOW with character and word-level n-grams. Experiments were carried out with and without stop words and the proposed approach has shown the best performance with stop words. The stacked model shows the best performance with 93.82% accuracy and 86.2% MCC which is better than existing models for Urdu fake news detection.

The current study does not employ deep learning methods, which is seen as a limitation of this study. In addition, only two ensemble approaches, bagging, and stacking have been utilized which necessitates additional experiments with further ensemble approaches. This study only focuses on TF-IDF and BoW feature computation. Other approaches such as word embedding, word2vec, and doc2vec have not been employed. The dataset is slightly imbalanced with a ratio of 65:35 for fake and real news. In the future, deep neural network models for Urdu fake news detection will be used. We also plan to extend the dataset further, which is suitable for deep learning models. We also intend to introduce embedding like pre-trained embedding, word embedding, FastText, *etc*. For dataset balancing, we plan to use GAN, SMOTE, or ADASYN approaches.

## Supplemental Information

10.7717/peerj-cs.1353/supp-1 Supplemental Information 1 Raw Data

10.7717/peerj-cs.1353/supp-2 Supplemental Information 2 Code for models

## Additional Information and Declarations

### Data Availability

The following information was supplied regarding data availability: The data and code are available in the Supplemental Files. The data is available at Zenodo: Imran Ashraf. (2023). Data for Urdu fake news. https://doi.org/10.5281/zenodo.7773474.
